# Spatial and Social Media Data Analytics of Housing Prices in Shenzhen, China

**DOI:** 10.1371/journal.pone.0164553

**Published:** 2016-10-26

**Authors:** Chao Wu, Xinyue Ye, Fu Ren, You Wan, Pengfei Ning, Qingyun Du

**Affiliations:** 1 School of Resources and Environmental Science, Wuhan University, Wuhan, China; 2 Key Laboratory of GIS, Ministry of Education, Wuhan University, Wuhan, China; 3 Key Laboratory of Digital Mapping and Land Information Application Engineering, National Administration of Surveying, Mapping and Geoinformation, Wuhan University, Wuhan, China; 4 Collaborative Innovation Center of Geospatial Technology, Wuhan University, Wuhan, China; 5 Department of Geography, Kent State University, Kent, Ohio, United States of America; University of Texas at San Antonio, UNITED STATES

## Abstract

Housing is among the most pressing issues in urban China and has received considerable scholarly attention. Researchers have primarily concentrated on identifying the factors that influence residential property prices and how such mechanisms function. However, few studies have examined the potential factors that influence housing prices from a big data perspective. In this article, we use a big data perspective to determine the willingness of buyers to pay for various factors. The opinions and geographical preferences of individuals for places can be represented by visit frequencies given different motivations. Check-in data from the social media platform Sina Visitor System is used in this article. Here, we use kernel density estimation (KDE) to analyse the spatial patterns of check-in spots (or places of interest, POIs) and employ the Getis-Ord $G_{i}^{*}$ method to identify the hot spots for different types of POIs in Shenzhen, China. New indexes are then proposed based on the hot-spot results as measured by check-in data to analyse the effects of these locations on housing prices. This modelling is performed using the hedonic price method (HPM) and the geographically weighted regression (GWR) method. The results show that the degree of clustering of POIs has a significant influence on housing values. Meanwhile, the GWR method has a better interpretive capacity than does the HPM because of the former method’s ability to capture spatial heterogeneity. This article integrates big social media data to expand the scope (new study content) and depth (study scale) of housing price research to an unprecedented degree.

## Introduction

Residential property is a multidimensional and durable commodity, and its value is determined by a combination of characteristics categorized as structural, locational and neighbourhood attributes [1–3]. However, these attributes do not have individual market prices. Numerous studies have explored the relationships between housing prices and specific attributes by exploring the implicit prices of attributes based either on the hedonic pricing(HPM) method [4] or the geographically weighted regression(GWR) method [5,6]. Analyses with either HPM or GWR can explicitly determine the market prices of specific factors by identifying the corresponding coefficients. Traditional location theories indicate that real estates located in proximity to commercial centres, green spaces and other facilities commands a higher margin price [7, 8]. Previous studies have generally measured ‘point of interest’ (POI) effects by calculating the distance or travel time between POIs and dwellings [9–16]. For instance, a commercial centre serves as a place of employment, entertainment, shopping and social contacting for most people. Intuitively, housing prices are expected to be higher near commercial centres. Similarly, green spaces provide a pleasing environment and improve the quality of life. Many scholars have revealed that green spaces exhibit value-added effects on housing prices [8, 17–19]. However, the presence of commercial centres and green spaces only has an effect over a certain range, and these effects vary across space. Traditional methods that treat commercial centres and green spaces as points or use landmarks to replace these POIs are not appropriate or objective. The preferences that determine whether an individual will visit a commercial centre or green space are influenced by distance considerations as well as the popularity and relative activity of the space. Therefore, commercial centres and green spaces can be classified as ‘hot spots’ and ‘cold spots’ depending on the number of visitors and the sites’ clustering patterns, with hot-spot commercial centres and green spaces exhibiting good development and comparatively high visit frequencies. These two categories can be used to characterize the ability of commercial centres or green spaces to attract visitors and assess the economic conditions and environmental quality of the surrounding areas. Hot spots are spatial clustering areas for certain types of social or economic activity and represent a preponderance of activity, which allows them to provide a greater number of desired resources and services. Conversely, the clustering of these locations may also result in negative impacts, such as traffic, noise pollution and security challenges. The presence of hot spots and housing price are likely correlated, although few studies have examined this possibility.

The process of collecting statistics on the number of people who access a POI is difficult to implement. Even if such statistics can be determined, the results may be inaccurate, and the data collection procedure may be expensive and time consuming. Therefore, we propose a method for analysing social media data to determine the spatial patterns of POI usage. With the development of Information Communication Technology (ICT) and location-based services (LBS), the use of Web 2.0 applications such as public social networks and enterprise social networks [20–22] for content creation and exchange have become prevalent. In this article, we focus on public social networks, which can provide data to analyse the behaviours of the public. Social media data can be used as a representative data of big geospatial data [23] and to provide business and academic communities with an unprecedented opportunity to study and analyse urban areas [24, 25], human behaviour [26], user identification [27], and popular sentiments regarding such areas. High-profile social media platforms, such as Twitter, Facebook and Sina, provide users with the ability to share their location and activity status information (often called ‘check-in’ data) in real time. Check-in data record a user’s activity information, which is represented by words, photos and expressions, at specific spots or POIs at a given time. Neuhaus [28] used Twitter data and presented preliminary descriptions of urban landscapes. Wakamiya [29] utilized social networks as a mirror of the public’s perceptions of the real world and measured crowd activity on Twitter, and he used these data to describe urban areas. In addition, he noted that people tend to live near places considered popular and convenient. Martinez [30] suggested that Twitter could be used to measure public sentiments about a given urban environment. Frias-Martinez [31] hypothesized that social media data offer an important indicator of the interactions between individuals and their environments. Noulas [32] and Frias-Martinez used social media data to study land uses. Shen examined the connections between different types of land uses by utilizing social media check-in data [33]. In previous studies, landscapes [34], urban areas [35], neighbourhood environments [36] and land uses [37, 38] all showed significant effects on housing prices. These efforts suggest that open-access ‘big data’ can illustrate the variations in sensibilities and preferences with regard to location evaluations. However, in addition to the analysis of human mobility based on social media data [26,39], few studies have used social media data mining to measure how public opinion on a location affects housing prices, which is an issue salient to people’s livelihoods. Soo [33] measured opinions on housing by quantifying the qualitative tone of local news media coverage of housing issues, thereby. In this way, Soo (2013) developed an approximated understanding of using social media to study housing price. Moreover, check-in data have the potential to represent human sentiment and POI attractiveness. In addition, according to the literatures [26, 40, 41], check-in data have the merit of representing and indicating the purposes of people with demand-tags associated with check-in activities, tracking the movement of people in a city. Therefore, we classify the POIs based on the motivation of travelling and the tags of POIs.To some extent, users’ check-in behaviours are comparable for the same motivation (the same type of POI) and can reflect the opinions and geographical preferences of individuals. In this article, we use the number of check-ins at each POI to represent the residents’ perceptions of that POI’s quality and activity. The use of check-in data replaces attempts to count the number of visitors, which is a method used in traditional statistical studies.

Furthermore, we supplement traditional housing price data sources by exploiting open-access social media data. However, identifying the appropriate scale at which a GWR model of housing prices should operate is not a straightforward task. Several studies on housing prices use parcels as their units of analysis [42]. In this article, the housing unit is applied as the unit of analysis, which has been infrequently implemented in previous studies. Choosing the housing unit as our unit of analysis allows us to identify the influence of internal attributes on housing prices and requires the ability to analyse big data. Using crawler technology, we retrieve housing attribute data from the real estate website SOFANG [4]. Following previous studies, we select green spaces (GRE) and commercial and business facilities (CBF) as the types of POI hot spots and test whether CBF and GRE hot spots fulfil specific functions in urban areas and whether their presence influences housing prices. A novel aspect of this study is the connection made between the real world and the virtual world using check-in data to study housing prices from a big-data perspective. This study uses Shenzhen, China as its case study and detects hot spots through the use of check-in data collected from July 2014 to June 2015. We use the terms ‘hot spot’ and ‘cold spot’ to describe the degree of measured activity across Shenzhen. As examples, we select POIs (CBF and GRE) that previous studies have reported as having an effect on housing prices. Finally, this article explores the effects of the degree of activity in CBF and GRE on housing prices using the framework of the GWR method. This article provides an evaluation of the effects of CBF and GRE on housing prices by analysing publicly accessible social media check-in data. This article introduces social media check-in data into the study of housing prices and provides new perspectives for future research. Using ubiquitous big social media data, we can better reveal the internal mechanisms that drive housing prices and public purchasing decisions by combining traditional housing price data with open-access social media data.

The remainder of this article is structured as follows. Section 2 briefly introduces the study area, housing dataset and check-in data. Section 3 provides a detailed description of the statistical method, Getis—Or $G_{i}^{*}$ (employed to detect hot spots using check-in data) and the framework of the GWR, which we use to evaluate factors that influence housing prices. Section 4 presents a discussion of the hot-spot and cold-spot statuses of different types of POI, and GWR is applied to explore the relationship between the various factors and housing prices. Finally, section 5 summarizes the conclusions of this study. The data analysis tools used in this article are MATLAB R2012a, ArcGIS 10.2, pgAdmin and SPSS 19.

## Materials

### Case study: Shenzhen

**Fig 1:**
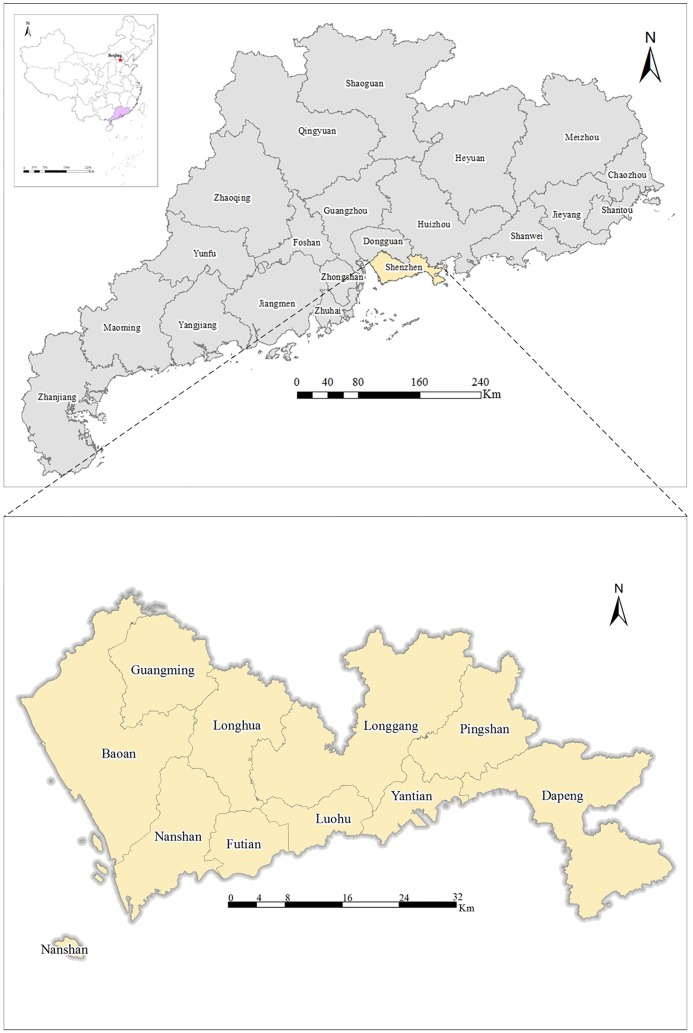
The geographical location of the case study city: Shenzhen.

Shenzhen is one of the most important cities in South China, with a land area size of 1,996.85 million km^2^ and a population of 10.78 million (as of 2014). The city is composed of the districts of Luohu, Futian, Nanshan, Longgang, Bao’an, Yantai, Guangming, Longhua, Pingshan and Dapeng (Fig 1). Since China initiated reforms and the ‘opening-up’ process, Shenzhen has served as China’s window to the world. With increasing economic development, housing prices in Shenzhen have risen and have become the subject of increasing attention. Moreover, Jones Lang LaSalle named Shenzhen one of the world's most dynamic cities in 2016. Therefore, this city offers a good case study for exploring the influence of urban hot spots on housing prices because the levels of POI activity (measured with check-in data) change dramatically during the same period in which housing prices are increasing.

### Housing price dataset

We choose Shenzhen as our case study and collect new housing transaction data from Shenzhen Research Centre for Digital City Engineering for 27,112 dwelling units (totalling 159 real-estate properties) from July to December 2015. The data for certain housing and real estate attributes are obtained from the SOFANG website using crawler technology [43]. These attributes include apartment area, floor level, number of bedrooms, number of washrooms, green space ratio and plot ratio. Other locational and neighbourhood attributes, such as accessibility to the central business district (the Citizen Centre, which is the landmark of Shenzhen’s CBD), subway stations, bus stations, schools and hospitals are calculated by GIS and based on a network analysis. To avoid potential biases, this study limits the types of properties studied to ordinary commercial housing; duplex apartments and cottages are excluded from the study. Because of the short time span described by the trading data, it is reasonable to ignore the influence of time on housing prices.

Prior to the model estimation, the data are pre-processed (via data cleaning and collinear data processing) to discard abnormal values. We obtain 25,323 effective samples and then employ a collinearity inspection between variables. We identify 17 variables that influence housing prices and classify the variables into three types: structural variables, locational variables and neighbourhood variables. The descriptive statistics, variable description and expected effect signs are presented in Table 1.

**Table 1:** Measurement methods and housing characteristic variable signs.

	Variable	Variable definition and measurement methods	Mean	Std.dev.	Expected sign
Structural Variables	Area	Square footage of the living area of house (m^2^)	100.487	35.689	+
	Floor	Floor on which the unit is situated (floor)	18.018	10.067	+
	NumBed	Number of bedrooms in unit	3.139	0.965	+
	NumWash	Number of bathrooms in unit	1.725	0.643	Unknown
	Fee	Property management fees (RMB)	3.868	1.069	+
	RPlot	Ratio of floor area	3.976	1.404	-
	RGreen	Ratio of green space area (%)	0.345	0.0729	+
Locational Variables	DCBD	Distance to CBD (central business district; km)	23.339	10.208	-
	DSub	Distance to nearest metro station (km)	0.319	0.409	-
	DBus	Distance to nearest bus station (km)	0.019	0.023	-
	DHospital	Distance to nearest hospital (km)	0.122	0.786	Unknown
	DSnursery	Distance to nearest nursery school (km)	0.597	0.464	-
	DSprimary	Distance to nearest primary school (km)	0.756	0.464	-
	DSmiddle	Distance to nearest middle school (km)	0.108	0.640	-
	DPark	Distance to nearest park(km)	0.113	0.609	-
Neighbourhood Variables	GiZScore_G	Degree of activity in green space	0.438	2.308	-
	GiZScore_C	Degree of activity in commercial centre	1.181	3.229	-

### Check-in data

Check-in data serve as typical crowd-sourcing geographic data (CSGD) [44]. Users can tweet check-in data using a GPS device (such as a smartphone or tablet) to record their location at a given time. Check-in data are geo-tagged information that includes the check-in time and social information. Members of the public use check-in data to record their daily lives; therefore, these data can be used to reflect the average person’s daily activities. We select check-in data recorded from the social media platform Sina Visitor System between July 2014 and June 2015. The data are primarily related to the various types of POIs, and user and positional data are the most important units of information. Considering the randomness of check-in behaviour, we pre-process the data to delete superfluous and invalid records, as well as misbehaving or fake users [45]. We filter the check-in data using the following critera (i) the location of a check-in is not in Shenzhen based on the geographical location; (ii) the name of the POI is not correct based on the text discriminant; (iii) we believe that users who have only one check-in record are not valid users and that their behaviours are accidental in a given time period(July 2014 and June 2015 in this article); (iiii) and based on the third criterion, we filter valid POIs that are signed by at least one valid user. Finally, we acquire 447,778 check-in records from 216,165 users and 22,670 POI. To ensure the privacy of personally identifiable information (PII) [46], we do not identify the information of users. This article uses 13,268 CBF sites and 1,413 GRE sites identified by researching the locations and names of POIs based on the Code for Classification of Urban Land Use and the Planning Standards of Development Land [47]. The CBF includes commercial facilities, business facilities, entertainment and sports facilities, and public utility outlets, and the GRE consists primarily of parks, green belts and urban squares. POI types and aggregated information can be seen in Table 2.

**Table 2:** POI types and aggregated information.

Type	Abbreviation	Counts	Percentage
Commercial and business facilities	CBF	13,268	59%
Industrial	IND	890	3.93%
Transport facilities	TRA	1,696	7.48%
Residence communities	RES	3,569	15.74%
Green space	GRE	1,413	6.23%
Administration and public services	APS	1,834	8.09%
Total		22,670	100%

## Methods

### Spatial hot-spot analysis method based on check-in data

In this section, we present our proposed method for using check-in data to detect hot spots. Each check-in record includes the user ID, time, coordinates (longitude and latitude), POI ID, and name and category of the venue. We use address-coding technology to locate records for our database and detect hot spots using the number of check-ins, which are obtained from POI data [48]. The POI data include the POI ID, coordinates, check-in totals, user counts and categories. We measure the hot spots by identifying their relationship with different types of POIs and exploring the effects of CBF and GRE.

Before detecting the hot-spot status of the different types of POIs, we analyse the overall spatial patterns. The kernel density estimation (KDE) is used to analyse the spatial distribution of all check-in POI data. KDE attempts to produce a smooth density surface of spatial point events in geographic space [49]. The general form of a kernel density estimator is given by

$$f\left(s\right)=\sum_{i=1}^{n}\frac{1}{\pi h^{2}}k\left(\frac{d_{is}}{h}\right)$$ (1)

where *f*(*s*) is the KDE function at location *s*, *h* is the bandwidth of the KDE, *d*_*is*_, is the distance from point *i* to location *s*, and the function *k* (called the kernel function) represents the spatial weight function of the ratio between *d*_*is*_ and the bandwidth *h*. Briefly, the results of the KDE rely on kernel sizes (called the search radius) and grid sizes. In this analysis, first, individual output cells were partitioned in 100m*100m areas, which can provide greater precision in the estimation most efficiently. Because too small of a grid size can increase the computational cost, whereas too large of a size can result in important details being ignored. Second, we select a search radius of 1000 m based on multiple testing rounds. Thresholds of less than 1000m, such as 600m and 800m, make the density distribution focus on a limited extent of elemental points, and the overall characteristic are not obvious. Conversely, thresholds of greater 1000m, such as 1200m and 1500m, make the density distribution overly smooth, and local differences cannot be well represented. We set 1000 m as the ideal threshold because it is the longest distance that remains comfortable to travel on foot (10–15 minutes) and obtains relatively good results in this paper.

**Fig 2:**
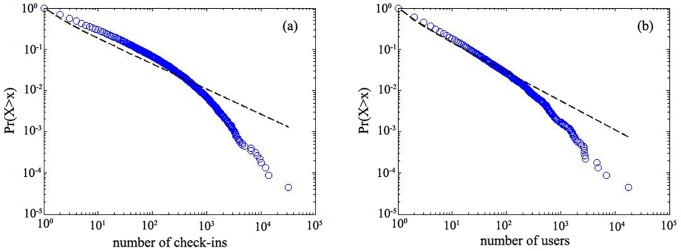
The power-law distribution patterns of check-ins (a) and users (b).

After kernel density analysis, we rank the POIs based on the counts of check-ins and users for all check-in spots to detect the POIs that are popular. The power-law distribution patterns (Fig 2) show that there are far more POIs with fewer check-ins and users than POIs with many check-ins and users. The patterns are similar to those in a previous study [33]. The results of Fig 2 suggest that POIs with high popularity, represented by the numbers of check-ins and users, provide more attractions to residents and are better known to people. Differences in peoples’ preferences and usages for POIs reveal that the formation of hot spots and cold spots for POIs is reasonable. We apply the ‘average nearest neighbour’ index to judge whether the spatial distribution of points is clustered or dispersed. This process is conducted for GRE and CBF separately. The clustering patterns of GRE and CBF are used to determine whether they are hot spots.

Because of the discrete distribution of points and simplification of the amount of big data [50, 51], we use grids to replace points to analyse the spatial patterns [52]. First, we divide the study area into 500 m x 500 m spaces and then apply the grid. Testing determined that 500 m is the optimal choice because a grid size larger than 500 m tends to mask local differences and because a grid size smaller than 500 m can exaggerate local characteristics. We then calculate the total number of check-in records and the number of POIs for each grid. We explore the possible hot spots using the grids and then use Getis-Ord $G_{i}^{*}$ to identify the hot spots. The Getis-Ord $G_{i}^{*}$ statistical clustering method [53, 54] is used to detect significant clusters of high value within a city according to locations that contain many people. Getis-Ord $G_{i}^{*}$ is a local statistic used to assess each feature within the context of neighboring features. This article defines a ‘hot spot’ as a place with a high degree of activity. The statistics are calculated as follows:

Eq 2 for a feature *i*:

$$G_{i}^{*}=\frac{\sum_{i=1}^{n}w_{ij}x_{i}-\bar{X}\sum_{i=1}^{n}w_{ij}}{S\sqrt{\frac{\left[n\sum_{i=1}^{n}w_{ij}^{2}-{\left(\sum_{i=1}^{n}w_{ij}\right)}^{2}\right]}{n-1}}}$$ (2)

where *x*_*i*_ represents the attribute value of *j*, *w*_*ij*_ represents the spatial weight of *j* and *i*, and n represents the total number of features.$\bar{X}$ and *S* are calculated according to Eqs 3 and 4, respectively.

$$\bar{X}=\frac{\sum_{i}^{n}x_{i}}{n}$$ (3)

$$\text{S}=\sqrt{\frac{\sum_{i=1}^{n}x_{i}^{2}}{n}-{\left(\bar{X}\right)}^{2}}$$ (4)

**Fig 3:**
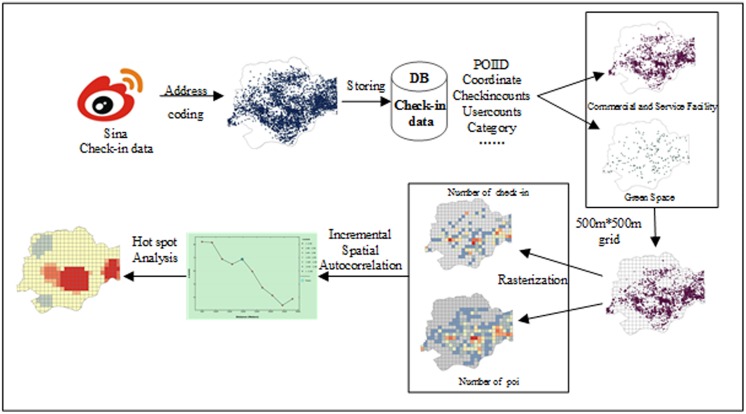
Spatial hot-spot analysis model based on check-in data.

Using these equations, we can obtain the z-score for each grid. For positive z-scores that are statistically significant, a larger z-score indicates a more intense clustering of high values (which identifies a hot spot) and vice versa. It is necessary to choose a method for conceptualising the spatial relations prior to the hot spot analysis. This article compares the results of various methods such as the inverse distance, fixed-distance band, Delaunay triangulation and space-time window methods. We select the fixed-distance band method to conceptualize the spatial relations because it is a good option for polygonal features. An appropriate value for the fixed-distance threshold is vital. This article applies incremental spatial autocorrelation to obtain z-score peaks that reflect the distances that correspond to the most pronounced spatial clustering processes [55]. Fig 3 uses Futian, Shenzhen as an example to illustrate the process in detail.

### Geographically weighted regression

GWR was introduced by Fotheringham [6] and is an extension of HPM, which is based on ordinary least squares (OLS) [4]. The essence of the HPM is a multiple linear regression (MLR) analysis. The attribute coefficients can be interpreted as the percentage change in housing prices for the semi-logarithmic function [56]. GWR is a spatial regression technique that takes spatial non-stationarity into consideration and allows local parameters to be estimated. The model is expressed as Eq 5:

$$\text{y}=\beta_{0}(u_{i},v_{i})+\sum_{k}\beta_{k}(u_{i},v_{i})X_{ik}+\varepsilon_{i}\;\;i=1,\ldots \ldots ,\text{n}$$ (5)

where (*u*_*i*_, *v*_*i*_) represents the coordinates (longitude, latitude) of observation *i*, *β*_0_(*u*_*i*_, *v*_*i*_) represents the intercept value, *β*_*k*_(*u*_*i*_, *v*_*i*_) represents the estimated parameter for the kth variable of observation *i* and varies for different locations, and *ε*_*i*_ represents the error term. The GWR method is superior to the HPM because of its ability to capture spatial heterogeneity. The parameter *β*_*k*_(*u*_*i*_, *v*_*i*_) is estimated as follows:

$$\hat{\beta }\left(u_{i},v_{i}\right)={\left(X^{T}W\left(u_{i},v_{i}\right)X\right)}^{-1}X^{T}W\left(u_{i},v_{i}\right)Y$$ (6)

where the weighting matrix is a diagonal matrix and the off-diagonal elements are all zero. Namely, *W*(*u*_*i*_, *v*_*i*_) = *dia*(*W*_*i*1_, *W*_*i*2_ … … *W*_*ij*_ … … *W*_*in*_). The geographical weightings of observation *i* and observation *j* are represented by *W*_*ij*_. In this study, we obtain weighting matrixes for all the observations by using a fixed Gaussian kernel function: $W_{ij}=\text{exp}\left(d_{ij}^{2}/h^{2}\right)$. In addition,$d_{ij}=\sqrt{{\left(u_{i}-u_{j}\right)}^{2}+{\left(v_{i}-v_{j}\right)}^{2}}$ is the Euclidean distance between *i* and *j*, and *h* is a non-negative parameter (bandwidth) that represents the decay degree with distance. An appropriate bandwidth can be selected based on the minimum Akaike information criterion for the GWR model (AICc) [57].

## Results and Discussion

### Spatial dynamic change in Shenzhen

**Fig 4:**
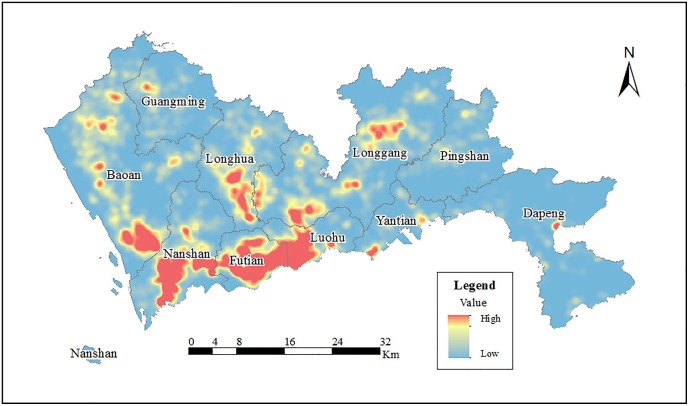
Kernel Density Estimation analysis of POI check-in data.

We use KDE to analyse the spatial distribution characteristics for all POI check-in data. As shown in Fig 4, the areas shaded in red indicate greater kernel density, greater activity frequency and greater concentrations, whereas the areas shaded in blue denote lower kernel density, smaller activity frequency and a reduced relative dispersion. It is no surprise that the largest clusters in Fig 4 are located in Luohu, Futian and Nanshan and at Luohu-Futian centre and Qianhai centre because these are the most prosperous areas in Shenzhen. The larger clusters are in the centres of Longhua and Longgang. The results of our kernel density analysis indicate that the clustered pattern of check-in POIs is not the result of chance.

For the average nearest neighbour statistic, the null hypothesis states that features are randomly distributed. The nearest neighbour ratios are 0.56 and 0.32 (both less than 1) for GRE and CBF, respectively, which indicates that the patterns exhibit clustering. We obtain z-scores of -31.30 and -149.64 for GRE and CBF, respectively, which indicates that less than 1% of their clustered pattern could be the result of random chance. Detailed information on the average nearest neighbour statistic is shown in Table 3. The spatial distribution of the POI check-in data presents high concentrations, thereby indicating the concentrated geographic interest preference of residents in Shenzhen. The results of the average nearest neighbour analysis for different types of POI provide valuable information for detecting hot spots.

**Table 3:** Average nearest neighbour summary.

Values	GRE	CBF
**Observed Mean Distance:**	455.3936 Meters	89.5055 Meters
**Expected Mean Distance:**	806.4292 Meters	278.8823 Meters
**Nearest Neighbour Ratio:**	0.564704	0.320943
**z-score:**	-31.303063	-149.637195
**p-value:**	0.000000	0.000000

### Hot-spot and cold-spot status for different types of POIs

**Fig 5:**
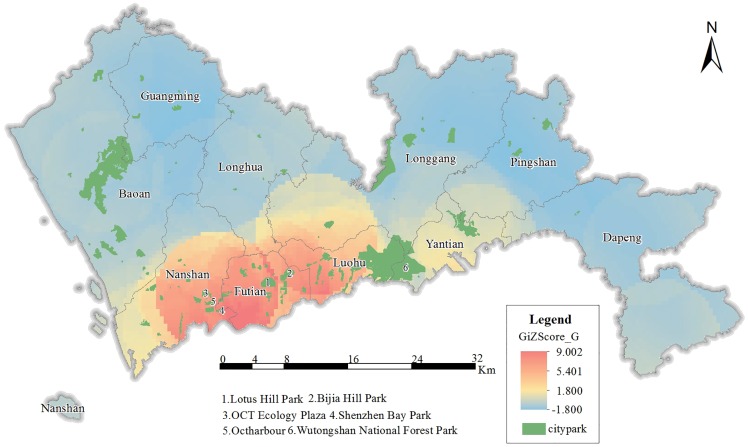
Hot spot distribution of GRE.

Fig 5 shows the hot spot distribution of GRE. Overall, the GRE hot spots are mainly distributed in Futian, Nanshan, Yantian and Luohu. The most significant clusters are in Lotus Hill Park (Label 1), Bijia Hill Park (Label 2), Wutongshan National Forest Park (Label 6), Octharbour (Label 5), Shenzhen Bay Park (Label 4) and OCT Ecology Plaza(Label 3). Lotus Hill Park is located in Futian’s centre and serves as a green ‘background’ to Shenzhen’s CBD; it is famous for its bronze statue of Deng Xiaoping and attracts many visitors. Bijia Hill Park is not far from Lotus Hill Park and is located adjacent to the Huaqiangbei commercial district; its visit frequency is high. Wutongshan National Forest Park is a bastion of natural beauty located near the border between Luohu and Yantian, and Mount Wutongshan is the highest mountain in Shenzhen. Octharbour attracts nearby residents for its peculiar wetland park. Shenzhen Bay Park has 12 different theme parks in total and is famous for its unique Mangrove forests. The OCT Ecology Plaza is located within an urban residential community and urban green space. This plaza plays the role of community centre and is a vital and popular site.

**Fig 6:**
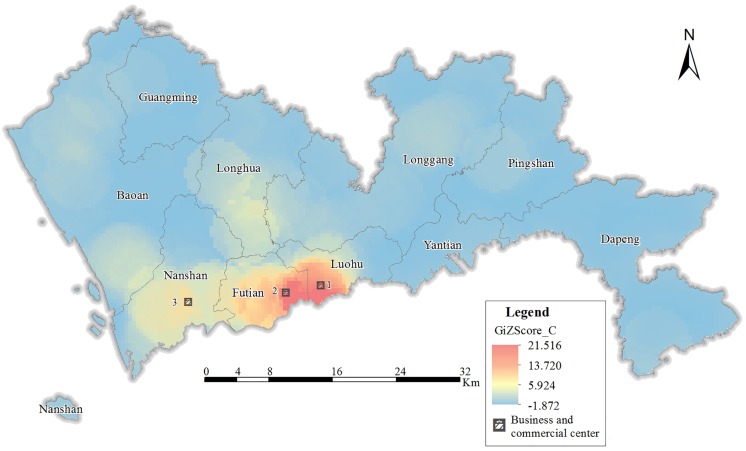
Hot spot distribution of CBF.

Fig 6 shows that CBF hot spots are mainly concentrated in the Dongmen commercial circles (Label 1), Huaqiangbei central business district (Label 2) and Nanshan central business district (Label 3). Dongmen has a long history as Shenzhen’s traditional commercial centre, and its food services and businesses reflect the characteristic culture of Shenzhen, thereby attracting many visitors. The Huangqiangbei commercial area has been successfully transformed from an industrial zone into a prosperous business area and was named the No. 1 Street for Chinese electronic production at the 16th China Hi-Tech Fair in 2008. The Nanshan central business district is an emerging business district in Shenzhen and serves the business needs as well as the expositional and cultural needs of the public. These districts all exercise significant influence on the residents of the surrounding areas. Additionally, the Bao’an and Longgang districts also show a certain degree of concentration, although it is not high.

### Analysis of effects on housing prices

#### Hedonic price method results

This article uses a typical hedonic equation for housing prices in a semi-logarithmic form (ln [price]) to analyse the influence of specific attributes on housing prices. This analysis allows us to identify the significant factors, and a semi-logarithmic form is used instead of a nonlinear form because it can better represent the relationship between housing prices and influential factors. Additionally, the semi-logarithmic form provides more accurate results compared with the linear form. The estimated parameters are calculated based on OLS. The results are presented in detail in Table 4. The F-value of the global model is significant at 1%, and the goodness of fit is 0.724. Therefore, the results of the global model are statistically significant, and the explanatory variables of the HPM account for 72.4% of the housing price variance. Table 4 lists the variables that are significant at 1% according to a t-test. The Variance Inflation Factors (VIFs) of all the variables are less than 10, which indicates that multicollinearity does not occur among the variables. It is notable that the activity degrees and clustering degrees of the CBF and GRE both have positive effects on housing prices. Specifically, housing prices surged by 8.3% with each unit increase in the CBF clustering degree. Similarly, a significant positive correlation is observed between the GRE clustering degree and housing prices, with an increase in the GRE clustering degree of 1 resulting in a 13.4% increase in housing prices. The distance to the CBD also displays significant effects on housing prices, and greater proximity to the CBD produces a higher price by 32.6%. The HPM assigns the most important attributes in the DCBD to the *t*-ratio (*t*-ratio = -55.863), which is consistent with the findings of previous studies [58–60]. The structural attributes, such as the number of washrooms, floor height, area, property management fees and green ratio, all have effects on housing prices and add 9.4%, 6.3%, 29.3%, and 2.3%, respectively, of extra value to the price of housing with a 1- unit increase. The effects of location variables are similar as expected with the exception of distance to primary schools. A 1- km increase in the distance to parks, hospitals, bus stations and subway stations reduces housing prices by 11.1%, 9.4%, 4.9% and 4.9%, respectively. Primary schools are an exception because of the policy of school district housing in China. The effect of school location on housing prices is determined by distance as well as school quality.

**Table 4:** Hedonic price method (HPM) parameter estimate summary.

Explanatory variable	Estimated coefficient	*t*-ratio	p-value
Constant	0.060	19.388	.000***
DCBD	-0.326	-55.863	.000***
Fee	0.293	43.604	.000***
DPark	-0.111	-27.376	.000***
NumWash	0.094	21.743	.000***
GiZScore_C	0.083	17.104	.000***
DHospital	-0.094	-24.942	.000***
SPrimary	0.080	21.966	.000***
Floor	0.063	20.077	.000***
GiZScore_G	0.134	24.466	.000***
DBus	-0.049	-14.273	.000***
DSub	-0.049	-11.417	.000***
Area	0.031	6.907	.000***
RGreen	0.023	6.854	.000***
R^2^	0.724		

*** Significant at the 1% level.

#### Geographically weighted regression results

**Fig 7:**
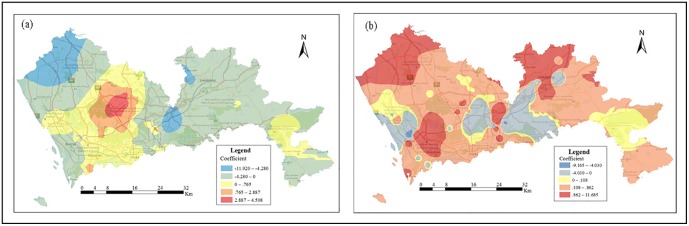
Spatial variation of the local parameters of the GWR. (a) and (b) are GiZscore_C and GiZscore_G, respectively.

The results of the local regression analysis (GWR) are summarized in Table 5. As previously mentioned, this method evaluates spatial variations in the relationships between the variables and housing prices. In this model, the *R*^2^ value increases from 0.724 (HPM) to 0.9399 (GWR), thereby showing that the GWR method has a better interpretive capability than the HPM. The GWR can explain 93.99% of the variation according to the *R*^2^ value. We also measure the spatial auto-correlation (Moran’s I index = 0.041, z-score = 3.86) by calculating Moran’s I index for the residuals of the GWR to ensure that the validity of the model for the residuals is random. Additionally, the AICc of the GWR is -74703.46207, and the bandwidth equals 0.337, which are less than that of the HPM (AICc = -36082.16912). The difference in the AICc of greater than 3 suggests that the GWR is more representative of reality [56]. Table 5 shows that all the variables are significant at 1% or 5% with the exception of DPark (p-value = 0.855). Additionally, the outstanding achievement of the GWR method is its ability to visualize local parameters. Fig 7 presents the GiZscore_C and GiZscore_G. Because of the discrete distribution of points, we use the inverse distance weighted (IDW) interpolation method to obtain a better visual effect. The output cell size of the IDW processes is set to 100 m, and a natural breaks (Jenks) classification is applied. Considering that the coefficients have positive and negative values, we set a zero value as a class limit to distinguish the positive and negative effects.

**Table 5:** Geographically weighted regression (GWR) parameter estimate summary.

Explanatory Variable	Minimum	Lower quartile	Median	Upper quartile	Maximum	p-value
Constant	-12.8186	-1.38137	-0.2675	0.04614	10.28509	.000***
Floor	-0.35291	0.031246	0.040169	0.056628	0.339561	.000***
NumBed	-0.66773	-0.02149	0.040887	0.128011	0.493299	.000***
NumWash	-0.64065	-0.0432	0.022523	0.06847	0.358014	.000***
Area	-0.68338	-0.01717	0.074358	0.142633	1.335995	.000***
RPlot	-10.9418	-0.5452	-0.1074	0.145695	1.900368	.000***
RGreen	-3.84742	-0.0312	0.089782	0.399657	1.858314	.000***
Fee	-2.68112	0.015945	0.25459	0.621551	4.052185	.000***
DSub	-12.302	-0.40319	-0.02085	0.563174	15.22164	.000***
DBus	-4.17797	-0.35394	-0.03028	0.509983	23.5197	.004**
DSnursery	-18.9376	-0.1124	-0.03278	0.158163	4.604339	.000***
DSprimary	-12.4242	-0.13258	0.017652	0.261109	2.282185	.000***
DSmiddle	-3.55686	-0.41095	0.017384	0.249635	14.10076	.000***
DHospital	-4.868	-0.62254	-0.12827	0.300655	5.447187	.000***
DPark	-2.27624	-0.07367	0.012992	0.328673	11.93626	.855
DCBD	-16.3229	-0.74499	-0.09705	0.430907	1.94271	.000***
GiZScore_G	-11.9198	-2.41877	-0.1252	0.179357	4.508242	.000***
GiZScore_C	-9.16523	0.015567	0.463302	2.229864	11.68491	.000***
R^2^	0.9399					
Bandwidth	0.337					

***, ** Significant at the 1% and 5% levels, respectively.

The regression coefficient for GiZscore_C is greater than zero because major concentrations are observed in Futian, Longhua, central and northeast Nanshan, the southern part of Bao’an and Guangming and central Dapeng. The CBF is more strongly clustered, which is associated with more check-ins, and the housing prices are higher in these locations, which represent the most prosperous areas of each district and have more complete facilities and more workplaces. These areas provide convenient amenities to housing buyers, such as employment, cafes, shopping and entertainment. Therefore, the housing prices are high. However, Fig 7(a) presents unexpected results for Longgang, Luohu and Yantian, with lower housing prices despite the greater clustering (with more check-ins) of the commercial and business facilities. GiZscore_C has a negative effect on housing prices, and because of the development of Futian and Nanshan, the position of Luohu has been declining. Luohu is an old urban district that is currently facing the problem of an old-age demographic bump. Senior citizens tend to choose housing in locations with a good environment characterized by peace and quiet. The clustering of residents can cause noise and traffic jams, which may affect the residents’ daily lives and rest. Yantian has relatively poor traffic conditions and no subway system. Thus, the clustering of CBF in Yantian may result in worse traffic conditions. Similarly, GiZscore_C has a negative effect on housing prices in Yantian. Longgang is a relatively poorly developed area in Shenzhen and has comparatively low housing prices. Housing buyers in Longgang pay more attention to transportation and the convenience of getting to work. Thus, it is not difficult to imagine that GiZscore_C would have a negative effect on housing prices in Longgang.

The GWR model reveals that the relationship between GiZscore_G and housing prices is not stationary across the study area, as illustrated in Fig 7(b). Most major areas show a positive effect of GiZscore_C on housing prices, with the exception of the south of Bao’an, the west and centre of Longgang, and most of Yantian. Yantian was the first ‘national biome’ in South China, and the air quality is good, which attracts many people. However, Yantian’s traffic conditions are relative poor, and it is located in a fault zone in Shenzhen. Therefore, it is likely that people prefer to visit this scenic spot for recreational purposes rather than live there.

## Conclusions

This article suggests a framework for applying a GWR model and a big data perspective for evaluating the influence of urban hot spots on housing prices in Shenzhen, China. This work contributes to the field of housing price research by identifying POI hot spots through the analysis of check-in data and by exploring the influence of a POI’s degree of activity (from the perspective of popular perception) on housing prices. Despite the widespread use of crowd-sourced geographic data (CSGD) [49, 61–64], few studies have used these data to examine housing prices, which are closely tied to public opinion and sentiments. We apply GWR method to test the hypothesis that hot spots (identified using check-in data) have significant effects on housing values. Our results are consistent with expectations. In this article, we introduce the use of check-in data, which represent a novel data source in the field of housing price research. Additionally, we take spatial heterogeneity into consideration and explore the factors that influence housing prices for different locations. Finally, we chose Shenzhen, China as our case study because housing prices in Shenzhen have skyrocketed in recent years, which is an area of continued concern [61]. This article integrates big social media data and expands the scope (new research content) and depth (study scale) of housing price research to an unprecedented degree.

This article applies the Getis-Ord $G_{i}^{*}$ technique to detect hot-spot areas of CBF and GRE. We then explore the relationship between housing prices and hot spots using hedonic price modelling and geographical regression modelling. The *R*^2^ of the HPM is 0.724, which indicated that the HPM can be used to analyse the effects of hot spots from a global perspective. CBF and GRE hot spots both increase housing prices. The *R*^2^ of the GWR is 0.9399, which indicates a stronger interpretive capability compared with the HPM. The results of the GWR reveal that the effects of influential factors vary over space, as represented by different coefficients. Finally, we select typical and special areas for our interpretation.

Most of the data values require innovative methods of analysis to identify their potential uses [65]. Although this article provides a new perspective for the application of big social media data to the study of housing prices, it still has several limitations.

1. The scope of the housing price samples is small, and this article only addresses spatial heterogeneity and neglects temporal heterogeneity.
2. There are demographic biases among social media users, and the elderly and children may represent missing data, which may cause some deviations in the results.
3. We only explore hot spots for commercial service industry sites and GRE; other types of POI hot spots may also have positive or negative effects on housing prices.
4. We use only a year of check-in data and housing price data. The ability to process, reduce and mine the increasing volume of big data is still a challenge [66, 67].

Several of these limitations may be overcome with the wider use of social media data for housing price research. In future research, to avoid the bias of check-in data and to process big data effectively, we will continue to fully exploit multimedia data [51] by integrating social media data, cellular signal data, smart card data and taxi GPS data to reveal potential factors affecting housing prices in a big data environment [68]. We also tend to use the method proposed by [69] to mine the spatial and temporal relations of comments from websites such as Sofang to better understand buyers’ attitudes towards houses. Beyond that, we will use geographically and temporally weighted regression (GTWR) [70, 71] to model spatial and temporal heterogeneity [42] effects simultaneously in real estate market data.

## Supporting Information

S1 File Check-in data of Shenzhen. (XLSX)

S2 File Attributes of house. (XLSX)
